# Search for DQ2.5 and DQ8 alleles using a lower cost technique in patients with type 1 diabetes and celiac disease in a population of southern Brazil

**DOI:** 10.1590/2359-3997000000282

**Published:** 2017-06-23

**Authors:** Marília D. Bastos, Thayne W. Kowalski, Márcia Puñales, Balduíno Tschiedel, Luiza M. Mariath, Ana Luiza G Pires, Lavínia S. Faccini, Themis R. Silveira

**Affiliations:** 1 Universidade Federal do Rio Grande do Sul Porto Alegre RS Brasil Programa de Pós-Graduação em Saúde da Criança e do Adolescente, Universidade Federal do Rio Grande do Sul (UFRGS), Porto Alegre, RS, Brasil. Universidade de Santa Cruz do Sul (Unisc), Santa Cruz do Sul, RS, Brasil.; Universidade de Santa Cruz do Sul Santa Cruz do Sul RS Brasil; 2 Departamento de Genética UFRGS Porto Alegre RS Brasil Departamento de Genética, UFRGS, Porto Alegre, RS, Brasil; 3 Instituto da Criança com Diabetes Hospital da Criança Conceição Porto Alegre RS Brasil Instituto da Criança com Diabetes, Hospital da Criança Conceição, Porto Alegre, RS, Brasil; 4 UFRGS Porto Alegre RS Brasil Programa de Pós-Graduação em Saúde da Criança e do Adolescente, UFRGS, Porto Alegre, RS, Brasil; 5 Departamento de Genética UFRGS Porto Alegre RS Brasil Programa de Pós-Graduação em Saúde da Criança e do Adolescente, UFRGS, Porto Alegre, RS, Brasil. Departamento de Genética, UFRGS, Porto Alegre, RS, Brasil. Instituto Nacional de Genética Médica Populacional, Porto Alegre, RS, Brasil; Instituto Nacional de Genética Médica Populacional Porto Alegre RS Brasil; 6 UFRGS Porto Alegre RS Brasil Programa de Pós-Graduação em Saúde da Criança e do Adolescente, UFRGS, Porto Alegre, RS, Brasil. Hospital da Criança Santo Antônio, Porto Alegre, RS, Brasil.; Hospital da Criança Santo Antônio Porto Alegre RS Brasil

**Keywords:** Celiac disease, type 1 diabetes mellitus, HLA, genetic polymorphism

## Abstract

**Objective:**

To evaluate the frequency of DQ2.5 and DQ8 alleles using the Tag-single-nucleotide polymorphism (Tag-SNP) technique in individuals with type 1 diabetes mellitus (T1DM) and celiac disease (CD) in southern Brazil.

**Materials and methods:**

In a prospective design, we performed the search for DQA1*0501 and DQB1*0201 alleles for DQ2.5 and DQB1*0302 for DQ8 through Real-Time Polymerase Chain Reaction (RT-PCR) technique, using TaqMan Genotyping Assays (Applied Biosystems, USA). The diagnosis of CD was established by duodenal biopsy and genotypic determination performed by StepOne Software v2.3. Allelic and genotypic frequencies were compared between groups using Chi-square and Fisher’s exact tests and the multiple comparisons using Finner’s adjustment.

**Results:**

Three hundred and sixty two patients with a median age of 14 years were divided into 3 groups: T1DM without CD (264); T1DM with CD (32) and CD without T1DM (66). In 97% of individuals with T1DM and CD and 76% of individuals with CD without T1DM, respectively, the alleles DQ2.5 and/or DQ8 were identified (p < 0.001). DQ2.5 was more common in individuals with CD (p = 0.004) and DQ8 was more common in individuals with type 1 diabetes (p = 0.008).

**Conclusions:**

The evaluation of the alleles for DQ2.5 and DQ8 by Tag-SNP technique showed a high negative predictive value among those with T1DM, similar to that described by the conventional technique. The high frequency of DQ8 alleles in individuals with T1DM did not allow differentiating those at higher risk of developing T1DM.

## INTRODUCTION

Celiac disease (CD) is a chronic and permanent enteropathy caused by intolerance to gluten proteins of wheat, rye, barley in genetically-predisposed subjects (1). The immunological base of CD results from an imbalance of the innate and adaptive immune systems. In these conditions, gliadin, the main toxic component of gluten, crosses the intestinal epithelium activating the adaptive immune system and determining an increase in intestinal permeability. Peptides contained in gluten go through the lamina propria, where they can be de-starched by the tissue transglutaminase (TTG) enzyme. Such peptides are presented by HLA class II molecules (DQ2 and DQ8), which promote the activation of tissue inflammation effector cells seen in CD: CD4 helper T lymphocytes (2). The combination of alleles will determine a higher or lower risk of developing the diseases. The alleles DQB1*0201 and DQA1*0501 form haplotype DQ2.5; the alleles DQB1*0302 and DQA1*0301 form haplotype DQ8. There are also combinations DQA1*0201 and DQB1*0202 the form haplotype DQ 2.2 and alleles DQA1*0505 and DQB1*0301 that form haplotype DQ7. In the case of CD, the higher-risk combinations are DQ2.5 and DQ8 or DQ2.5 in homozygous form (3).

There is wide variation in the prevalence of CD in different countries. In Europe and the United States, the prevalence varies between 1 and 3% in the general population (4). The prevalence of CD confirmed by biopsy, in studies performed in Brazil to date, show a variation of 0.15 to 1.94% (5).

Individuals with type 1 diabetes have a higher prevalence of CD. A recent systematic review described a prevalence rate between 1.6 and 16.4% of CD among individuals with T1DM and recommended screening after the age of two years in this population (6). In Brazil, CD screening in patients with T1DM based on the serology is recommended, as the prevalence is considered similar to that of European countries and the United States (7,8).

HLA genes play an important role in autoimmune diseases such as T1DM and CD and its identification in individuals with such diseases is very important to understand susceptibility aspects, as well as different clinical presentations. The genotyping is carried out by methods based on Polymerase Chain Reaction (PCR) using Sequence-Specific Probes (SSO) with sequence specific primers (SSP) or Sequence-Based Typing (SBT). These traditional genotyping techniques involve many reactions, which makes them complex and expensive.

Monsuur and cols. (9) validated a technique using Tag single-nucleotide polymorphisms (Tag SNP), allowing the performance of tests with high sensitivity and specificity, at a lower cost. Brandao and cols. (10) evaluated patients with T1DM in northeastern Brazil and compared the costs of a conventional technique (SSP) with the technique using Tag SNP and observed an average cost of US$ 90-100 per person for the conventional technique, and US$ 5 per subject analyzed with the Tag SNP technique. Considering these results, the authors recommend that this genotyping method be used instead of the conventional technique, thereby reducing the overall costs of genetic identification for T1DM in areas with limited financial resources.

The HLA system is very polymorphic and displays variability between different geographical areas and ethnic groups. Brazil is a country with a high degree of miscegenation and great racial variation between regions. A recent study identified African ancestry in 50% of the northeastern population and 70% of European ancestry in the south and southeast regions of the country (11). There are no studies on the search for HLA DQ2 and DQ8 genes among individuals with T1DM and CD in the state of Rio Grande do Sul, Brazil.

The assessment of the frequency of HLA-DQ types in individuals with T1DM and/or CD is interesting because both diseases have an autoimmune etiology, where the presence of HLA (Human Leukocyte Antigen) class 2 molecules represents the main genetic risk factor (12,13). Among the identified risk alleles, we highlight HLA-DQA1*05 and DQB1*02 on chromosomes 6p21, which can estimate a high negative predictive value for CD, but a low positive predictive value, since 35 to 40% of the population usually have one or both alleles (14,15).

CD screening in patients with T1DM is recommended, aiming at reducing both the morbidity of T1DM and the consequences of untreated CD, even if it is asymptomatic (16,17). Consequently, with the genetic investigation of these groups, we hope to rationalize the performance of high-cost tests that would not help in the identification of the groups with the concomitant diseases.

The aim of this study was to evaluate the frequency of alleles for DQ2.5 and DQ8 using the Tag SNP technique in individuals with T1DM and CD, in a population of southern Brazil.

## MATERIALS AND METHODS

### Study design and population

A prospective study was carried out from August 2012 to October 2014, involving individuals diagnosed with type 1 diabetes treated at *Instituto da Criança com Diabetes* (ICD) – *Hospital da Criança Conceição*, located in Porto Alegre, the state capital of Rio Grande do Sul (RS) – Brazil and individuals diagnosed with CD, confirmed by duodenal biopsy, residents in the city of Porto Alegre, RS – Brazil, participating in *Associação dos Celíacos do Brasil – Rio Grande do Sul* (ACELBRA-RS).

Blood and/or saliva samples were collected from individuals with T1DM, when undergoing periodic assessment for diabetes control and after providing authorization to participate in the study. Among these individuals, we identified those who had TTG-IgA < 9.0 U/mL or TTG-IgA > 16 U/mL and duodenal biopsy classified as ≥ 2, according to Marsh criteria (18) modified by Oberhuber (19). The following individuals were excluded from the study: those with TTG-IgA values between 9.0 U/mL and 16 U/mL; TTG-IgA values > 16 U/mL that did not undergo duodenal biopsy or those in whom Marsh classification was < 2.

Authorization was requested from individuals with a diagnosis of CD to participate in the study during an event sponsored by ACELBRA-RS. A saliva sample was collected and an interview was carried out to identify those who had the diagnosis confirmed by biopsy. Individuals who did not undergo duodenal biopsy and those whose diagnosis was doubtful were excluded from the study.

### Laboratory methods

#### DNA extraction

A peripheral blood sample (10 mL of whole blood with anticoagulant) was collected for subsequent DNA extraction using the salting out method (20). When unable to collect blood, a saliva sample was collected using the Oragene kit (DNA Genotek^®^) and submitted to DNA extraction according to the manufacturer’s instructions.

#### Haplotypic Determination of HLA-DQ2.5 and -DQ8

The prediction of haplotypes HLA-DQ2.5 and HLA-DQ8 was carried using the Tag Single nucleotide polymorphism (Tag-SNP) technique (9,10). The Real-Time Polymerase Chain Reaction technique (RT-PCR) was performed through an assay by TaqMan^®^ Genotyping Assays (Applied Biosystems, USA) according to the manufacturer’s instructions. The assays used were previously deposited in Custom TaqMan Genotyping Assay (Applied Biosystems) and included: C_58662585_10 (*rs2187668* C>T of *HLA-DQA1*) and C_29817179_10 (*rs7454168* C>T of *HLA-DQB1*). The analysis of results and the genotypic determination of polymorphisms was performed using StepOne Software v2.3 (Applied Biosystems). The DQ8 and DQ2.5 haplotypes were predicted from the identified genotypes, as described by Monsuur and cols. (9) and Brandao and cols. (10).

## Statistical analyses

Statistical analyses were performed using the Statistical Package for Social Sciences (SPSS), version 22.0 (IBM Corp. – Armonk, NY, USA) and R, version 3.2.2 (R core team, 2015). The chi-square test or Fisher’s exact test were used to compare the frequencies of haplotypes HLA-DQ2.5 and DQ8 between the groups. In situations involving multiple comparisons, Finner’s adjustment was used for significant values (P), with significance being set at p < 0.05.

## Ethical considerations

The study was developed according to the rules of the National Health Congress based on Resolution 466/12 and was approved through Brazil Platform (CAE 01260412.7.0000.5347) by the Research Ethics Committee of the following institutions: *Grupo Hospitalar Conceição*, where the data collection from individuals with T1DM was carried out and *Universidade Federal do Rio Grande do Sul* (UFRGS), a committee associated to the institution where the research originated (Postgraduate Program in Child and Adolescent Health – UFRGS). Upon protocol completion, patients or their caregivers were asked to provide authorization for study participation by reading and signing the free and informed consent form.

## RESULTS

A total of 362 patients were evaluated, divided into 3 groups: individuals with T1DM and negative antibodies for CD (group 1 = 264 individuals), individuals with T1DM and with CD diagnosis (group 2 = 32 individuals) and people with CD without a diagnosis of T1DM (group 3 = 66 individuals).

Table 1 shows the distribution of the combined haplotypes of DQ2.5 and DQ8 in the 3 groups, in which there is the presence of at least one DQ2.5 or DQ8 allele in 97% of individuals in the group with T1DM and CD (group 2) and in 76% in individuals with CD without a diagnosis of T1DM (group 3) (p < 0.001). It was also observed that the combination DQ2.5/DQx was more frequent in group 3, whereas the combination DQ2.5/DQ8 was more frequent in group 2 and the absence of alleles DQ2.5 and/or DQ8 occurred more frequently in group 3.

**Table 1:** Distribution of combined DQ2.5 and DQ8 haplotypes

HLA Haplotypes	Group 1 T1DM without CD n (%)	Group 2 T1DM with CD n (%)	Group 3 CD without T1DM n (%)
DQ2.5/DQ2.5	23 (8.7)	7 (21.9)	9 (13.6)
DQ2.5/DQX^a^	50 (18.9)^1^	9 (28.1)	28 (42.4)^1^
DQ2.5/DQ8	71 (26.9)^2^	10 (31.3)^3^	5 (7.6)^2,3^
DQ8/DQ8	16 (6.1)	0 (0.0)	1 (1.5)
DQ8/DQX^a^	58 (22.0)	5 (15.5)	7 (10.6)
DQX^a^/DQX^a^	46 (17.4)^4^	1 (3.1)^4,5^	16 (24.2)^5^
Total	264 (100)	32 (100)	66 (100)

n: number of analyzed individuals. ^a^ DQX: non-DQ2.5 or DQ8 haplotypes; p < 0.001.

1, 2, 3, 4 and 5 Finner’s adjustment for multiple comparisons with p < 0.05.

The presence of DQ2.5 alleles occurred in 170 (57.4%) individuals with T1DM (Groups 1 and 2), being more frequent in the group diagnosed with CD (group 2). The presence of DQ8 alleles occurred in 160 (54.1%) individuals with T1DM, and there was no difference between the groups with or without CD (Table 2).

**Table 2:** Presence of DQ2.5 and DQ8 alleles among individuals with T1DM

HLA	Group 1 T1DM without CD n (%)	Group 2 T1DM with CD n (%)	p
DQX^a^	120 (45.5)	6 (18.8)	0.004
DQ2.5	144 (54.5)	26 (81.2)	
DQX^a^	119 (45.1)	17 (53.1%)	0.388
DQ8	145 (54.9)	15 (46.9%)	

n: number of analyzed individuals. a DQX: non-DQ2.5 or DQ8 haplotypes.

When comparing individuals with T1DM and a CD diagnosis (group 2) and individuals with CD without a diagnosis of T1DM (group 3) no significant difference was observed between groups regarding DQ2 alleles. However, individuals in group 2 had a significantly higher frequency of DQ8 allele (Table 3).

**Table 3:** Presence of DQ2.5 and DQ8 alleles among individuals with T1DM and CD and among those with CD and without T1DM

HLA	Group 2 T1DM with CD n (%)	Group 3 CD without T1DM n (%)	p
DQX^a^	6 (18.8)	24 (36.4)	0.102
DQ2.5	26 (81.2)	42 (63.6)	
DQX^a^	17 (53.1)	53 (80.3%)	0.008
DQ8	15 (46.9)	13 (19.7%)	

n: number of analyzed individuals. a DQX: non-DQ2.5 or DQ8 haplotypes.

Table 4 shows the comparison between individuals with T1DM without CD (Group 1) and those with a diagnosis of CD, regardless of the presence of T1DM (groups 2 and 3). It was observed that the presence of the DQ2.5 allele is more frequent in patients with CD, while the DQ8 allele is more frequent in the group that has T1DM.

**Table 4:** Presence of DQ2.5 and DQ8 alleles among individuals with T1DM without CD and among those with CD irrespective of the presence of T1DM

HLA	Group 1 T1DM without CD n (%)	Groups 2 and 3 CD n (%)	p
DQX^a^	120 (45.5)	30 (30.6)	0.011
DQ2.5	144 (54.5)	68 (69.4)	
DQX^a^	119 (45.1)	70 (71.4)	< 0.001
DQ8	145 (54.9)	28 (28.6)	

n: number of analyzed individuals. a DQX: non-DQ2.5 or DQ8 haplotypes.

## DISCUSSION

The performance of genotyping using the Tag SNP technique has been validated in individuals with T1DM and CD, being considered effective and less costly, allowing the performance of population screening studies (9,21,22). Our study aimed to perform the genotyping of HLA DQ2.5 and DQ8 using the Tag SNP technique in individuals with T1DM and individuals with CD in a population of southern Brazil, which confirmed the high negative predictive value of the test in the group with T1DM. Megiorni and Pizzuti (23), in a review on the practical implications of identifying HLA risk alleles in individuals with CD, reaffirmed the importance of the negative tests as a more significant value.

A study carried out in Italy in 1005 patients with CD, used the Tag SNP technique to genotype haplotypes DQ2.5, DQ8, DQ2.2 and DQ7, comparing it with the traditional technique by PCR-SSP and obtained high sensitivity and specificity, recommending it to be used in population screenings and suggesting studies in other population groups (24). A more recent study carried out in Brazil with DNA extracted from 329 umbilical cord blood samples, compared the two techniques, using the same haplotypes of the present study and concluded that the results obtained by real-time PCR are highly reliable, with no discordant results when compared to the PCR-SSP technique (25).

Megiorni and cols. (26) established a risk gradient for CD based on HLA DQ and also defined the combination of haplotypes DQ2 and DQ8 as higher risk. Gutierrez-Achury and cols. (27) performed an extensive genetic study in patients from the United States, England, and the Netherlands with concomitant T1DM and CD, with T1DM without CD and with CD without T1DM and concluded that genotype DQ2.5/DQ8 shows an increased risk of concomitant disease. We found that 27% of individuals with T1DM (Group 1) and 31% of individuals with T1DM and CD (Group 2) had this combination of haplotypes, whereas in the group of individuals with CD without T1DM (Group 3), the concomitant haplotypes occurred in 7.6%, with this group showing a higher frequency of individuals with only DQ2.5 alleles.

Individuals with T1DM, at any age, have a higher risk of CD, but because both diseases are associated with HLA DQ genotypes, the search for HLA-DQ2 and DQ8 is not always useful for identifying predisposed groups (28). The difference in frequency of DQ8 alleles disclosed in Table 4, confirms this difficulty in finding risk alleles for CD in the three groups. We found that the alleles for DQ2.5 were more prevalent among individuals with CD; however, the DQ8 alleles are more frequent in individuals with T1DM, which does not allow differentiating between the groups with and without CD.

The search for alternative techniques is based on the fact that the high negative predictive value of the test will help to reduce costs with the periodic investigation of patients who do not have the alleles, but also to reassure the patients or their relatives about the prospect of having the concomitant diseases. In Brazil, in 2009, the Federal Government (Official Gazette) published a statement recommending that T1DM patients should undergo CD screening through TTG-IgA at the start of T1DM and every year regardless of the clinical manifestations (29). When considering the cost-benefits of the genotype assessment of individuals with T1DM, we observed that the average cost of serological screening in our country is US$ 6 per person and should be performed annually, while the genotype assessment for each allele with the described technique costs US$ 5 per patient and will be performed on only one occasion.

Although we observed a high negative predictive value among individuals with T1DM and CD, it was observed that 24% did not show the haplotypes for DQ2.5 and DQ8 in the group with CD without T1DM. Karell and cols. (30) evaluated populations of different European countries and screened for the haplotypes DQA1*05 and DQB1*02 for DQ2 and DQA1*03 DQB1*0302 for DQ8 and found a heterogeneous distribution, with a higher prevalence of negative DQ2 and DQ8 in Italy when compared to France, Finland and England. Koskinen and cols. (31) evaluated risk haplotypes for CD in 3 countries and identified in the group of Italian patients the presence of alleles for DQ2.2 (DQB1*0202 and DQA1*0201) and DQ7 (DQB1*0301) by 27% and 18% of the population, respectively. Kotze and cols. (32), in a study carried out in southern Brazil, found a frequency of 8.9% of individuals with CD with DQ2 and/or DQ8 negative alleles, and warned for the high degree of miscegenation in the country.

Considering this study was carried out in a region of Brazil with high prevalence of Italian immigrants and taking into account the high rate of European ancestry seen in the south and southeast regions of the country (11), the possibility of a higher incidence of other DQ risk alleles, different from DQ2.5, should be considered. The search for DQA1*0501 and DQB1*0201 alleles allowed the identification of DQ2.5 individuals, but did not identify DQ2.2 and DQ7 individuals.

In conclusion, the search for DQ2.5 and DQ8 alleles using the Tag-SNP technique allowed us to obtain a high negative predictive value for the diagnosis of CD in a population with T1DM, similar to what is described in the literature using the conventional technique.

There was a high frequency of DQ8 allele in individuals with T1DM when compared to individuals with CD without T1DM. However, the presence of this allele in individuals with T1DM does not indicate an increased risk of CD in the assessed population.

Considering the high degree of miscegenation of the Brazilian population, we recommend the inclusion of the search for DQ2.2 and DQ7 alleles in the southern and southeastern regions of Brazil, to increase the sensitivity and specificity of CD risk investigation.

## References

[1] Schuppan D, Junker Y, Barisani D. Celiac disease: from pathogenesis to novel therapies. Gastroenterology. 2009;137(6):1912-33. 10.1053/j.gastro.2009.09.008 19766641

[2] Rubio-Tapia A, Murray JA. Celiac disease. Curr Opin Gastroenterol. 2010;26(2):116-22. 10.1097/MOG.0b013e3283365263 PMC2830645 20040864

[3] Liu E, Lee HS, Aronsson CA, Hagopian WA, Koletzko S, Rewers MJ, et al Risk of pediatric celiac disease according to HLA haplotype and country. N Engl J Med. 2014;371(1):42-9. 10.1056/NEJMoa1313977 PMC4163840 24988556

[4] Gutierrez-Achury J, Coutinho de Almeida R, Wijmenga C. Shared genetics in coeliac disease and other immune-mediated diseases. J Intern Med. 2011;269(6):591-603. 10.1111/j.1365-2796.2011.02375.x 21401738

[5] Diniz-Santos D, Machado A, Silva L. Doença Celíaca. In: Carvalho E de, Silva LR, Ferreira CT, editors. Gastroenterologia e Nutrição em Pediatria. São Paulo: Manole; 2012. p. 359-403.

[6] Pham-Short A, Donaghue KC, Ambler G, Phelan H, Twigg S, Craig ME. Screening for Celiac Disease in Type 1 Diabetes: A Systematic Review. Pediatrics. 2015;136(1):e170-6. 10.1542/peds.2014-2883 26077482

[7] Araújo J, da Silva GA, de Melo FM. Serum prevalence of celiac disease in children and adolescents with type 1 diabetes mellitus. J Pediatr (Rio J). 2006;82(3):210-4. 10.2223/JPED.1478 16729151

[8] Diniz-Santos DR. Doença celíaca em crianças e adolescentes com diabetes mellitus tipo1 Salvador, Bahia. Tese (Doutorado). Programa de Pós-graduação em Medicina e Saúde. Universidade Federal da Bahia; 2010.

[9] Monsuur AJ, de Bakker PIW, Zhernakova A, Pinto D, Verduijn W, Romanos J, et al. Effective detection of human leukocyte antigen risk alleles in celiac disease using tag single nucleotide polymorphisms. PLoS One. 2008;3(5):e2270. 10.1371/journal.pone.0002270 PMC2386975 18509540

[10] Brandao LC, Vatta S, Guimaraes R, Segat L, Araujo J, De Lima Filho JL, et al. Rapid genetic screening for major human leukocyte antigen risk haplotypes in patients with type 1 diabetes from Northeastern Brazil. Hum Immunol. 2010;71(3):277-80. 10.1016/j.humimm.2009.12.008 20035815

[11] Kehdy FSG, Gouveia MH, Machado M, Magalhães WCS, Horimoto AR, Horta BL, et al.; Brazilian EPIGEN Project Consortium. Origin and dynamics of admixture in Brazilians and its effect on the pattern of deleterious mutations. Proc Natl Acad Sci U S A. 2015;112(28):8696-701. 10.1073/pnas.1504447112 PMC4507185 26124090

[12] Tsouka A, Mahmud FH, Marcon MA. Celiac disease alone and associated with type 1 diabetes mellitus. J Pediatr Gastroenterol Nutr. 2015;61(3):297-302. 10.1097/MPG.0000000000000789 25806677

[13] Troncone R, Discepolo V. Celiac disease and autoimmunity. J Pediatr Gastroenterol Nutr. 2014;59 Suppl 1:S9-S11. 10.1097/01.mpg.0000450394.30780.ea 24979198

[14] Romanos J, Rosen A, Kumar V, Trynka G, Franke L, Szperl A, et al. Improving coeliac disease risk prediction by testing non-HLA variants additional to HLA variants. Gut. 2014;63(3):415-22. 10.1136/gutjnl-2012-304110 PMC3933173 23704318

[15] Wijmenga C, Gutierrez-Achury J. Celiac disease genetics: past, present and future challenges. J Pediatr Gastroenterol Nutr. 2014;59 Suppl 1:S4-7. 10.1097/01.mpg.0000450392.23156.10 24979196

[16] Sud S, Marcon M, Assor E, Palmert M, Daneman D, Mahmud F. Celiac disease and pediatric type 1 diabetes: diagnostic and treatment dilemmas. Int J Pediatr Endocrinol. 2010;2010:161285. 10.1155/2010/161285 PMC2905696 20652072

[17] Weiss B, Pinhas-Hamiel O. Celiac disease and diabetes: when to test and treat. J Pediatr Gastroenterol Nutr. 2017 Feb;64(2):175-9. 10.1097/MPG.0000000000001388 27574884

[18] Marsh MN. Gluten, major histocompatibility complex, and the small intestine. A molecular and immunobiologic approach to the spectrum of gluten sensitivity (‘celiac sprue’). Gastroenterology. 1992;102(1):330-54. 1727768

[19] Oberhuber G, Granditsch G, Vogelsang H. The histopatology of CD: time for standardized report scheme for pathologists. Eur J Gastroenterol Hepatol. 1999;11(10):1185-94. 10.1097/00042737-199910000-00019 10524652

[20] Lahiri DK, Nurnberger JI. A rapid non-enzymatic method for the preparation of HMW DNA from blood for RFLP studies. Nucleic Acids Res. 1991;19(19):5444. 10.1093/nar/19.19.5444 PMC328920 1681511

[21] Lavant EH, Agardh DJ, Nilsson A, Carlson JA. A new PCR-SSP method for HLA DR-DQ risk assessment for celiac disease. Clin Chim Acta. 2011;412(9-10):782-4. 10.1016/j.cca.2010.12.033 21219892

[22] de Bakker PIW, McVean G, Sabeti PC, Miretti MM, Green T, Marchini J, et al. A high-resolution HLA and SNP haplotype map for disease association studies in the extended human MHC. Nat Genet. 2006;38(10):1166-72. 10.1038/ng1885 PMC2670196 16998491

[23] Megiorni F, Pizzuti A. HLA-DQA1 and HLA-DQB1 in Celiac disease predisposition: practical implications of the HLA molecular typing. J Biomed Sci. 2012;19(1):88. 10.1186/1423-0127-19-88 PMC3482388 23050549

[24] Vatta S, Fabris A, Segat L, Not T, Crovella S. Tag-single nucleotide polymorphism-based human leukocyte antigen genotyping in celiac disease patients from northeastern Italy. Hum Immunol. 2011;72(6):499-502. 10.1016/j.humimm.2011.03.008 21513759

[25] Selleski N, Almeida LM, Almeida FC, Gandolfi L, Pratesi R, Nóbrega YK. Simplifying celiac disease predisposing HLA-DQ alleles determination by the real time PCR method. Arq Gastroenterol. 2015;52(2):143-6. 10.1590/S0004-28032015000200013 26039834

[26] Megiorni F, Mora B, Bonamico M, Barbato M, Nenna R, Maiella G, et al. HLA-DQ and risk gradient for celiac disease. Hum Immunol. 2009;70(1):55-9. 10.1016/j.humimm.2008.10.018 19027045

[27] Gutierrez-Achury J, Romanos J, Bakker SF, Kumar V, de Haas EC, Trynka G, et al. Contrasting the Genetic Background of Type 1 Diabetes and Celiac Disease Autoimmunity. Diabetes Care. 2015;38 Suppl 2:S37-44. 10.2337/dcs15-2007 PMC4582914 26405070

[28] Korponay-Szabó IR, Troncone R, Discepolo V. Adaptive diagnosis of coeliac disease. Best Pract Res Clin Gastroenterol. 2015;29(3):381-98. 10.1016/j.bpg.2015.05.003 26060104

[29] Diário Oficial da União. Poder Executivo. Brasília, Portaria Oficial da União; Poder Executivo, Brasília, DF, 18 de Setembro. Protocolo Clínico e Diretrizes Terapêuticas da Doença Celíaca. 2009. p. Seção I: 79-81.

[30] Karell K, Louka AS, Moodie SJ, Ascher H, Clot F, Greco L, et al. Hla types in celiac disease patients not carrying the DQA1*05-DQB1*02 (DQ2) heterodimer: results from the european genetics cluster on celiac disease. Hum Immunol. 2003;64(4):469-77. 10.1016/s0198-8859(03)00027-2 12651074

[31] Koskinen L, Romanos J, Kaukinen K, Mustalahti K, Korponay-Szabo I, Barisani D, et al. Cost-effective HLA typing with tagging SNPs predicts celiac disease risk haplotypes in the Finnish, Hungarian, and Italian populations. Immunogenetics. Immunogenetics. 2009;61(4):247-56. 10.1007/s00251-009-0361-3 19255754

[32] Kotze LM da S, Nisihara R, Utiyama SR da R, Kotze LR. Letters to the Editor. Rev Esp Enfermedades Dig. 2014;106(8):561-2. 25544420

